# Estimation of early life endogenous surfactant pool and CPAP failure in preterm neonates with RDS

**DOI:** 10.1186/s12931-019-1040-z

**Published:** 2019-04-16

**Authors:** Roberto Raschetti, Roberta Centorrino, Emmanuelle Letamendia, Alexandra Benachi, Anne Marfaing-Koka, Daniele De Luca

**Affiliations:** 1 Division of Pediatrics and Neonatal Critical Care, Medical Center “A.Béclère”, South Paris University Hospitals, AP-HP, Paris, France; 2 Division of Obstetrics and Gynecology, Medical Center “A.Béclère”, South Paris University Hospitals, AP-HP, Paris, France; 3 Division of Hematology, Medical Center “A.Béclère”, South Paris University Hospitals, AP-HP, Paris, France; 4 Physiopathology and Therapeutic Innovation Unit-UNSERM U999, South Paris-Saclay University, Paris, France

**Keywords:** Lamellar bodies, Preterm neonate, Respiratory distress syndrome, Continuous positive airway pressure

## Abstract

**Background:**

It is not known if the endogenous surfactant pool available early in life is associated with the RDS clinical course in preterm neonates treated with CPAP. We aim to clarify the clinical factors affecting surfactant pool in preterm neonates and study its association with CPAP failure.

**Methods:**

Prospective, pragmatic, blind, cohort study. Gastric aspirates were obtained (within the first 6 h of life and before the first feeding) from 125 preterm neonates with RDS. Surfactant pool was measured by postnatal automated lamellar body count based on impedancemetry, without any pre-analytical treatment. A formal respiratory care protocol based on European guidelines was applied. Clinical data and perinatal risk factors influencing RDS severity or lamellar body count were real-time recorded. Investigators performing lamellar body count were blind to the clinical data and LBC was not used in clinical practice.

**Results:**

Multivariate analysis showed gestational age to be the only factor significantly associated with lamellar body count (standardized β:0.233;*p* = 0.023). Lamellar body count was significantly higher in neonates with CPAP success (43.500 [23.750–93.750]bodies/μL), than in those failing CPAP (20.500 [12.250–49.750] bodies/μL;*p* = 0.0003).LBC had a moderate reliability to detect CPAP failure (AUC: 0.703 (0.615–0.781);*p* < 0.0001; best cut-off: ≤30,000 bodies/μL). Upon adjustment for possible confounders, neither lamellar body count, nor its interaction factor with gestational age resulted associated with CPAP failure.

**Conclusions:**

Early postnatal lamellar body count on gastric aspirates in CPAP-treated preterm neonates with RDS is significantly influenced only by gestational age. Lamellar bodies are not associated with CPAP failure. Thus, the endogenous surfactant pool available early in life only has a moderate reliability to predict CPAP failure.

## Background

Antenatal corticosteroid prophylaxis and early application of continuous positive airway pressure (CPAP) are considered the gold standard for, respectively, the prevention and treatment of respiratory distress syndrome (RDS) in preterm neonates [1, 2]. Surfactant replacement is recommended when CPAP fails [3, 4] and, if performed within the first 3 h of life, reduces death and/or bronchopulmonary dysplasia [5]. Surfactant is commonly administered based on inspired oxygen fraction (FiO_2_) [3], however, the suggested FiO_2_ thresholds are arbitrary, they can be reached beyond the optimal 3 h-time window and they lack of any physiopathological background. Moreover, contemporaneous measurement of oxygen saturation or PaO_2_ might be needed to accurately describe oxygenation. The CPAP level may also influence oxygenation, through its effect on the lung aeration. In fact, both recent pediatric and neonatal acute respiratory distress syndrome (ARDS) definitions include these variables in appropriate indexes [6, 7].

The available endogenous surfactant also influences lung aeration and, therefore, assessing the surfactant pool available early in life could be interesting to better understand the clinical picture and eventually guide surfactant replacement. Lamellar bodies are intracellular, easily measurable, surfactant storage granules released from type II-pneumocytes [8, 9]. Once in the alveolar space, lamellar bodies are subjected to the breathing cycle and form the surfactant layer adsorbed at the gas/liquid interface [9, 10]. Thus, lamellar body count (LBC) is an estimation of the available endogenous surfactant. LBC correlates with lung aeration evaluated by a semi-quantitative lung ultrasound score [11, 12]. LBC can be realized on amniotic fluid and is used as prenatal lung maturity test, as it has a good reliability to predict the RDS occurrence [13, 14], but is also doable postnatally (at the delivery or using gastric aspirates obtained before the first feeding) [15–17].

Despite this physiopathological background, the link between the available surfactant pool and the CPAP failure has never been investigated. Our purposes are: 1) to clarify what are the clinical factors affecting early postnatal LBC in CPAP-treated preterm neonates with RDS; and 2) to investigate reliability of postnatal LBC and its association to CPAP failure.

## Methods

### Patients

We designed a pragmatic, observational, blind, prospective cohort study. The study was conducted during 2014–2015 in an academic tertiary referral neonatal intensive care unit (NICU). All inborn preterm neonates (< 37 weeks’ gestation) with RDS treated with nasal CPAP were eligible for the study. In detail, RDS was diagnosed when the following criteria [6] were met: 1) occurrence of respiratory distress within the first 24 h of life; 2) presence of typical lung ultrasound or chest X-rays findings; [18, 19] 3) complete, sustained and prompt oxygenation improvement after surfactant replacement or significant improvement under CPAP which prevented surfactant administration; 4) no other respiratory disorders, as detailed below.

Exclusion criteria were: 1) chromosomal abnormalities or complex congenital malformations; 2) congenital lung diseases; 3) need for surgery in the first week of life;

4) early onset severe sepsis/septic shock, as defined elsewhere; [20] 5) transient tachypnea of the neonate, defined as mild (Silverman score ≤ 3) respiratory distress occurring in a neonate of more than 34 weeks’ gestation presenting with typical lung ultrasound or chest X-rays findings and resolving within the first 72 h of life; [6, 18, 19] 6) meconium aspiration syndrome, defined as the presence of meconium-stained amniotic fluid and airway secretions, occurrence of respiratory failure early from birth and typical lung imaging; [21] 7) blood aspiration syndrome, defined as respiratory distress and blood-stained amniotic fluid and airway secretions, onset of respiratory failure early from birth and typical lung imaging; [22] 8) pulmonary haemorrhage, defined as respiratory distress suddenly occurring together with bright blood-stained airway secretions, presence of left-to-right ductal shunting and typical lung imaging; [23] 9) any kind of neonatal ARDS, defined according to criteria described earlier [6].

All pregnancies received full prenatal care: gestational age was estimated based on the postmenstrual date and early gestation ultrasound findings; antenatal betamethasone was administered as two 12-mg doses 24 h apart, whenever delivery was expected to occur before 34 weeks. Steroid prophylaxis was considered complete when the second dose was given at least 24 h before the delivery. Small for gestational age (SGA) babies were classified according to Fenton’s curves [24]. Chorioamnionitis and obstetric cholestasis were clinically defined as described elsewhere [25, 26]. Gestational diabetes and pre-eclampsia were diagnosed as per current international guidelines [27, 28].

### Respiratory protocol

We use a formal respiratory care protocol derived from European guidelines for RDS management [3]. Delivery room intubation was performed only on persistently apnoeic or bradycardic babies, unresponsive to face-mask ventilation, according to international guidelines on neonatal resuscitation [29]. Neonates are started with variable or continuous flow-CPAP if they were ≤ or > 32 weeks’ gestation, respectively. This choice aims to reduce the work of breathing as much as possible in the smallest preterm neonates [30]. Appropriately sized nasal masks were used and particular care was applied to minimize CPAP interruptions. CPAP was set at 6 cmH_2_O and oxygen was added only if CPAP was not enough to maintain peripheral oxygen saturation within 90 and 95% [31]. Pacifiers of adequate size with drops of 30% glucose solution were used to reduce leaks and provide sedation, if needed [31]. CPAP was started in the delivery room just after stabilization or upon NICU admission for babies with gestational age ≤ or > 32 weeks, respectively [12].

CPAP failure was defined as need for surfactant replacement to treat RDS in the first 48 h of life. Surfactant was administered as poractant-α (Chiesi Farmaceutici, Parma, Italy; 200 mg/Kg), whenever FiO_2_ exceeded 0.3 or 0.4 for babies with gestational age ≤ or > 28 weeks’ gestation, respectively. The technique for surfactant administration, ventilation policy and use of sedation or caffeine have been described earlier [32]. All new residents and fellows are trained about the respiratory protocol every 6 months.

### Lamellar bodies measurement

We have chosen to use gastric aspirates, as this sampling is practically easier to perform than the amniotic fluid collection at the delivery. Moreover, specimens obtained through gastric aspirates are less likely to be contaminated by blood or meconium. Samples were collected upon the appearance of respiratory distress, within the first 3 h of life and anyway before the first feeding using a soft, end-hole suction catheter and applying a negative pressure of − 100 mmHg. Samples were collected in sterile polypropylene traps, immediately sent to the laboratory at room temperature and divided into two identical aliquots. The first aliquot was used for routine microbiological test and the second for LBC. We wanted to perform a pragmatic study in a large-volume perinatal centre, thus samples were not processed adding dithiothreitol or any other solvents, nor were they centrifuged or frozen. Samples were directly aspirated by automated cell counters (LH780, Beckman Coulter, Brea, CA – USA) and lamellar bodies were counted by impedancemetry through the platelet channel of the analyser, as previously described [9]. When the fluid was too viscous it was diluted with isotonic saline (1:1 *v*/v) and results were corrected for dilution. Investigators performing LBC were blinded to patients’ clinical conditions.

### Data collection

Basic clinical data and perinatal risk factors influencing the clinical severity of RDS or known to affect antenatal LBC were recorded in real time. LBC was recorded in a specific spreadsheet and merged with clinical data only at the end of recruitment, thus clinicians were blinded to LBC and they were not used for any clinical decision. Relevant NICU protocols did not change during the study. Participation to the study did not modify the common clinical care, since gastric aspirates culture is routinely used as microbiological screening in NICU-admitted babies (if obtained before the first feeding), according to our routine clinical protocol. The study was approved by local institutional review board (n.13–046) and consent was obtained from parents or guardian upon NICU admission.

### Statistics

Since this was the first study about LBC and CPAP failure, a formal sample size calculation was unfeasible: we continued the recruitment until we reached a sample size similar to that of other studies using the same technique [14]. Population was divided in the CPAP success or failure cohorts. Continuous data were tested for normality with Kolmogorov-Smirnov test, expressed as mean (standard deviation) or median [interquartile range] and then contrasted with Student or Mann-Whitney, accordingly. Spearman correlation was also performed between LBC and continuous variables. Proportions were compared with χ^2^ or Fisher test, as appropriate.

Multiple linear regression was finally applied on the whole population to identify factors affecting LBC. All variables potentially able to influence LBC were used as covariates. In detail, covariates included in the model were gestational age, antenatal steroids, clinical chorioamnionitis, obstetric cholestasis, 5’Apgar score and SGA status. In order to avoid multicollinearity, birth weight was not used, since it is highly correlated with gestational age (*rho* = 0.844;*p* < 0.0001). Enter technique was used and no covariates selection method was applied.

Receiver operating characteristic (ROC) analysis was used to evaluate the reliability of LBC to predict CPAP failure: area under the curve (AUC) and reliability data were reported with (95% confidence intervals). The relationship between LBC and CPAP failure was studied adjusting for possible confounders using a Poisson regression, with a logit link function and adjusted relative risks (_a_RR) were calculated. Gestational age, antenatal steroids, pre-eclampsia and LBC were inserted as covariates, since they had a *p* < 0.2 at the univariate comparison between CPAP success and failure cohorts. An alternative model was also tested including an interaction term between LBC and gestational age, as both these variables are significantly associated to RDS occurrence [33]. In order to avoid multicollinearity, birth weight was not used, since it is highly correlated with gestational age and SGA babies were not different between the two cohorts. Model goodness-of-fit was evaluated by Omnibus test. All analyses were performed with SPSS Version 15.0 (SPSS Inc., Chicago, IL, USA) and Medcalc 13.3 (MedCalc bvba, Ostend, Belgium). *p-*values < 0.05 were considered statistically significant.

## Results

We enrolled 192 neonates but in 67 LBC was not technically feasible due to the fluid viscosity (*n* = 50), meconium or blood contamination (*n* = 6) or insufficient volume (*n* = 11). Basic details and perinatal risk factors for RDS for the 125 neonates enrolled are described in Table 1: patients failing CPAP had lower gestational age, birth weight and tended to receive less antenatal steroids. CPAP failure occurred at 2.5 [1.5–4]h of life always appearing as increasing oxygen requirement and dyspnoea. No mother had hypothyroidism; no pneumothorax or pneumomediastinum were observed. Gastric aspirates were collected at 1 [0.5–2]h of life. There were no differences between neonates enrolled in the study and those for whom LBC was unfeasible for technical reasons (gestational age: 33.3 (2.9) weeks, *p* = 0.270; 5’Apgar: 9 (1.1), *p* = 0.593; antenatal steroids 61.2%, *p* = 0.195; caesarean delivery 56.7%, *p* = 0.999; SGA neonates: 6%,*p* = 0.10; CPAP failure: 28.4%, *p* = 0.627)).

**Table 1:** Basic population details and perinatal factors influencing RDS severity

Patients	Whole population (125)	CPAP success (85)	CPAP failure (40)	p
Gestational age (weeks)	31.6 (3.3)	32.9 (2.4)	29.3 (3.2)	**< 0.0001**
Birth weight (grams)	1641 (648)	1807 (593)	1327 (641)	**< 0.0001**
Complete antenatal steroids	89 (71%)	56 (66%)	24 (60%)	0.09
Clinical chorioamnionitis	7 (11%)	3 (3.5%)	4 (10%)	0.211
Gestational diabetes	2 (1.6%)	2 (2.3%)	0	0.999
Obstetric cholestasis	8 (6%)	6 (7%)	2 (5%)	0.962
Pre-eclampsia	21 (17%)	17 (20%)	4 (10%)	0.155
Premature rupture of membranes	10 (8%)	8 (9.4%)	2 (5%)	0.500
Caesarean section	71 (57%)	67 (79%)	33 (82%)	0.811
5′ Apgar score	8.9 (1.3)	8.9 (1.1)	8.8 (1.7)	0.529
SGA neonates	18 (14%)	13 (15%)	5 (12%)	0.660

Data are expressed as mean (standard deviation) or number (%). *p*-values refer to the comparison between CPAP success and failure cohorts with Student, χ^2^ or Fisher exact test, as appropriate

LBC was significantly higher in patients who received a complete course of prenatal steroids (38.000 [19.200–76.500] LBC/μL), than in those who received an incomplete course or no prophylaxis 23.000 [12.500–44.200] LBC/μL;*p* = 0.032), by the univariate analysis. LBC significantly correlated with gestational age (rho = 0.331;*p* < 0.001) and birth weight (*rho* = 0.844;*p* < 0.001). LBC did not differ between neonates born to mothers with or without pre-eclampsia (38.000 [12.000–96.500] vs 33.000 [18.000–69.000] LBC/μL;*p* = 0.764), clinical chorioamnionitis (25.000 [9000–45.000] vs 35.000 [18.500–74.000] LBC/μL;*p* = 0.171) or obstetric cholestasis (52.500 [10.500–69.750] vs 33.500 [18.250–71.500] LBC/μL;*p* = 0.764) and between neonates small or appropriate for gestational age (40.500 [26.250–134.250] vs 33.000 [15.000–68.250] LBC/μL;*p* = 0.169). LBC did not correlate with 5’Apgar score (*rho* = 0.024;*p* = 809). Multivariate analysis showed gestational age to be the only factor significantly associated with LBC (standardized β: 0.214; *p* = 0.034); all other covariates were removed from the model.

**Fig. 1:**
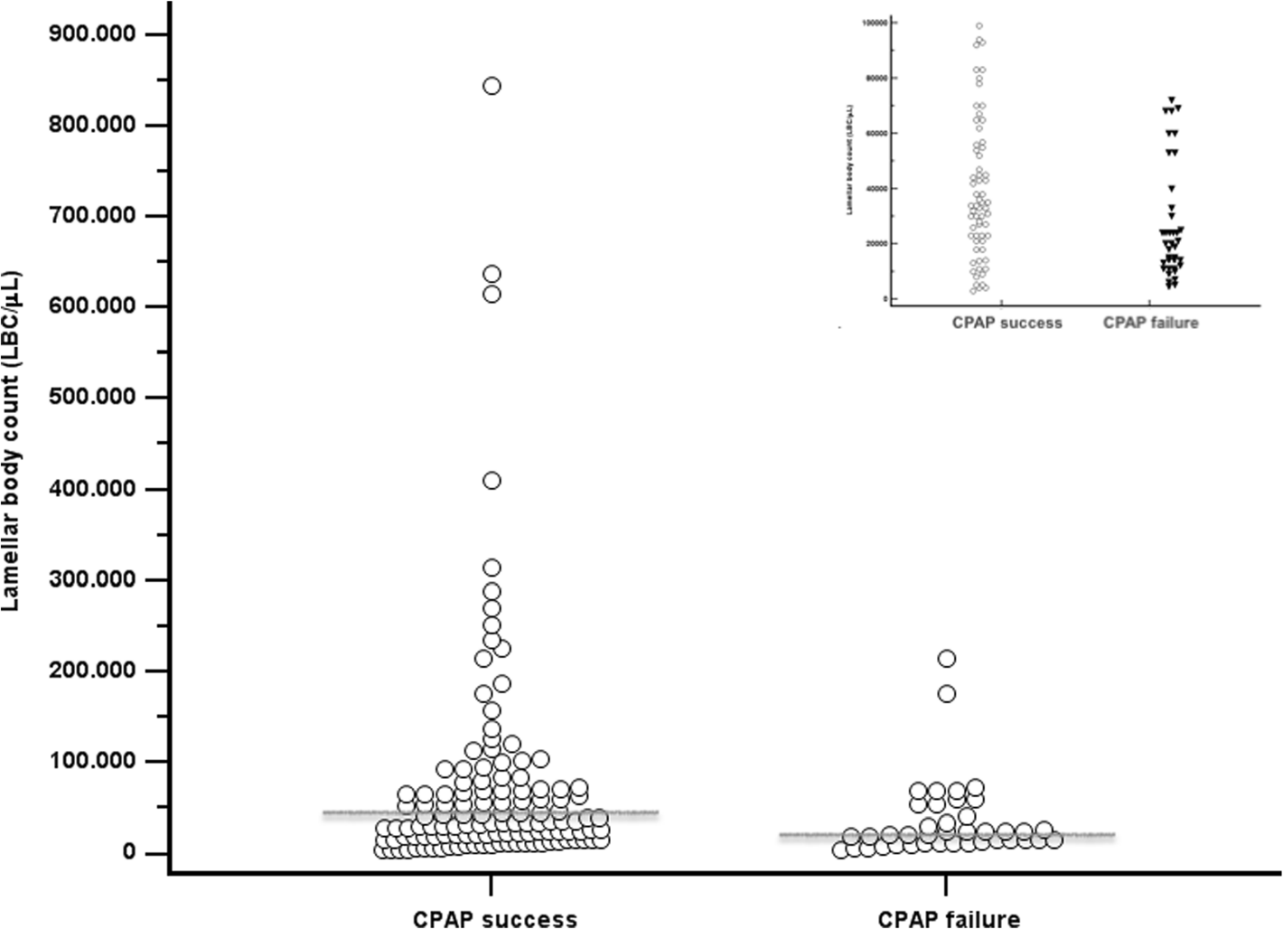
Lamellar body count (LBC) in CPAP success and failure cohorts. Data from all patients are depicted in the big picture, while the insert illustrates only data from patients with lamellar bodies are < 100,000 μL. Grey horizontal lines represent median values. LBC is higher in the CPAP success than in CPAP failure cohort (*p* = 0.0003; Mann-Whitney test); full data are reported in the text

Figure 1 shows that LBC was significantly higher in neonates with CPAP success (43.500 [23.750–93.750]LBC/μL), than in those failing CPAP (20.500 [12.250–49.750] LBC/μL;*p* = 0.0003). Subgroup analysis showed that neonates of gestational age ≤ 32 weeks with CPAP success (38,000 [22000–102,000]LBC/μL) had significantly higher LBC than those failing CPAP (20,000 [12000–30,000] LBC/μL; *p* = 0.008). Conversely, subgroup analysis for babies aged more than 32 weeks showed no significant differences (53,000 [18730–117,500] vs 45,000 [27000–92,000] LBC/μL; *p* = 0.845).

**Fig. 2:**
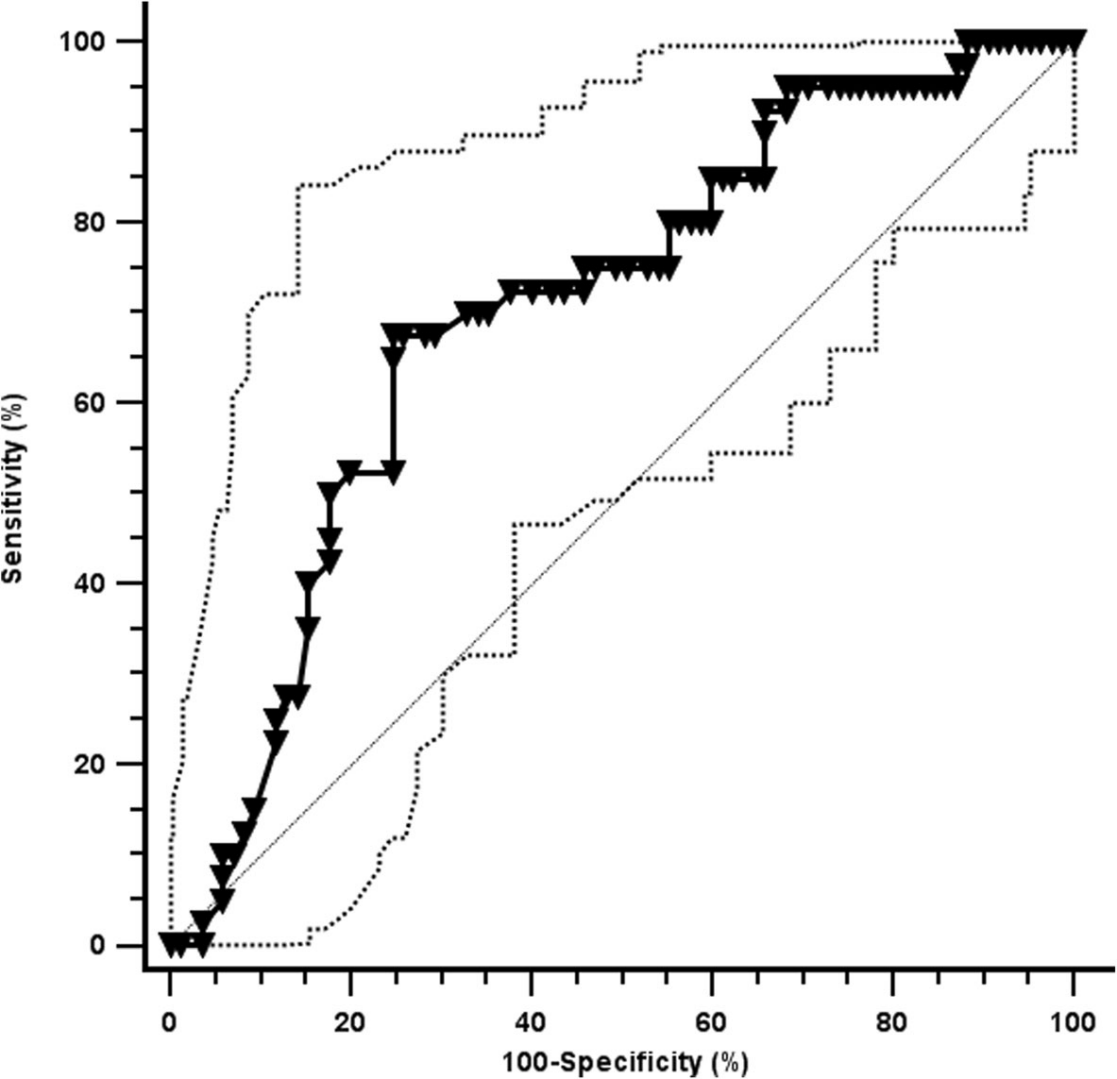
Receiver Operator Characteristic curve for the prediction of CPAP failure. Black and hatched lines represent the area under the curve (AUC) and its 95% confidence interval. Diagonal line represents an AUC of 0.5. More details in the text

ROC analysis is depicted in Fig. 2. LBC had a moderate reliability to detect CPAP failure (AUC: 0.703 (0.615–0.781);*p* < 0.0001; best cut-off: ≤30,000 LBC/μL; sensitivity: 70 (53.5–83.4)%; specificity: 67.1 (56–76.9)%; positive likelihood ratio: 2.1 (1.5–3.1); negative likelihood ratio: 0.45 (0.3–0.7); positive predictive value: 50 (36.3–67.3)%; negative predictive value: 82.6 (71.6–90.7)%). Reliability of LBC was not improved when performing a subgroup analysis for neonates of ≤32 weeks (AUC: 0.314 (0.181–0.447)) or > 32 weeks gestation (AUC: 0.473 (0.210–0.735)).

Upon adjustment for possible confounders (Table 2), gestational age and prenatal steroid prophylaxis resulted associated to CPAP failure, while LBC and the interaction factor ‘LBC*gestational age’ did not.

**Table 2:** Multivariate analysis results: factors associated with CPAP failure

	_a_RR for CPAP failure (95%CI)	*p*
*1st model*		
Gestational age	0.62 (0.51–0.76)	**< 0.0001**
Incomplete or no prenatal steroids	3.2 (1.07–9.6)	**0.037**
Pre-eclampsia	3 (0.7–12.2)	0.123
LBC	0.99 (0.9–1.004)	0.218
*2nd model (with interaction term)*		
LBC^*^Gestational age	1.001 (0.99–1.003)	0.213

Two models are presented: a first model including covariates with *p* < 0.20 at the univariate analysis and a second one also including an interaction term “LBC^*^Gestational age”. Goodness-of-fit is good for both models (*p* < 0.001, Omnibus test)

## Discussion

The knowledge of the available endogenous surfactant pool in RDS neonates undergoing CPAP might be an important information to understand the physiopathology and the clinical picture of each case. Moreover, if a point-of-care test measuring surfactant pool would be able to predict CPAP failure, that could theoretically help a more tailored and optimized surfactant therapy. We measured the early life surfactant pool in preterm neonates who received optimal perinatal care and were treated with CPAP according to formal protocols based on more recent evidences [1–3].

Our study shows that, in such patients, postnatal automated LBC (without any pre-analytical step): 1) is significantly influenced by gestational age, while other clinical factors do not seem to be relevant; 2) is not significantly associated with CPAP failure. Thus, the endogenous surfactant pool available early in life only has a moderate reliability to predict CPAP failure at any gestational age.

These two findings are novel as, to the best of our knowledge, LBC had never been tested in these conditions or aiming to predict CPAP failure in neonates with established RDS, while LBC has been used to predict RDS occurrence [14, 17, 33]. Our results might seem conflicting with experimental data showing increased LBC in lung tissue of animals treated with prenatal steroids. Nevertheless, we enrolled neonates with already established RDS according to clinical and imaging integrated data: thus, in our population, RDS occurred despite the eventual prenatal steroid prophylaxis, which did not produce a complete benefit. It is conceivable that, under these conditions, gestational age would be the only remaining variable significantly influencing LBC, since steroids failed to boost surfactant production enough to prevent RDS insurgence.

About the lack of LBC reliability to predict CPAP failure, ROC analysis suggests as best cut-off value the same (30.000 LBC/μL) that has been indicated to predict RDS occurrence, [14] although the reliability is much lower in our study. This may be related to the effect of CPAP. CPAP is known to be efficacious to treat RDS and spare surfactant replacement in preterm neonates [2]. The mean time needed for preterm neonates to produce enough endogenous surfactant is approximately 4 days [34] and meanwhile CPAP keeps the alveoli open improving the oxygenation, despite the relative surfactant deficiency. LBC only gives a picture of the surfactant pool available early after birth. Thus, the occurrence of RDS is more closely related to the initially available endogenous surfactant (that is, to LBC), than the clinical evolution overtime, which also depends on surfactant production along the days and to CPAP-induced alveolar recruitment. In fact, phosphatidylcholine concentration in fetal lung fluid does not correlate with clinical severity of RDS in preterm lambs [35]. Other factors influencing the clinical severity of RDS overtime (such as the degrees of alveolarization and lung tissue inflammation, type and level of CPAP, amount of extravascular lung water, nursing experience) may also have contributed to the reduced LBC reliability [36]. Multivariate analysis confirmed this issue, since the endogenous available surfactant pool, as measured by LBC, is not significantly associated to CPAP failure. This is fully consistent with the explanations suggested above, as the surfactant pool might impact on the RDS occurrence more than on its evolution [13, 14]. Conversely, modern perinatal care is likely to significantly impact on the course of RDS, as early CPAP is able to avoid surfactant treatment in a significant proportion of patients [37]. Subgroup analysis also confirms the relative lack of LBC reliability to predict CPAP failure at any gestational age and does not seem to suggest a particular patient subgroups where the technique could be more useful.

If we compare LBC to two other recently proposed tools to predict CPAP failure, such as the lung ultrasound score [12, 38] and the surfactant adsorption test (SAT), [11] in both cases LBC results inferior in terms of AUC. This can have several explanations. First, LBC is performed early from birth and CPAP failure may occur significantly later: a test performed significantly earlier tends to be less accurate than a test performed closer to the outcome to predict. Moreover, lung ultrasound score describes the lung aeration rather than the surfactant pool available, thus it takes into consideration the CPAP-induced alveolar recruitment and can also be serially repeated overtime. SAT is performed in the first hours of life similar to LBC, but it describes surfactant quality and properties, rather than its available amount. In fact, SAT measures surfactant adsorption and accumulation overtime at the air/liquid interface, for a given amount of phospholipids [11]. While the amount of available surfactant may be relevant for the RDS occurance, its quality and biophysical properties could be more important for the clinical severity, once RDS is established. In fact, surfactant adsorption significantly depends on surfactant-protein B, which is known to be related to the clinical severity of RDS [39].

We acknowledge some study limitations. More samples could have been assayed introducing additional analytical steps, such as solvent dilution or centrifugation/vortexing [16]. However, these steps could theoretically increase complexity and make the test less immediately available. Therefore, automated LBC without any pre-analytical steps might not be always pragmatically suitable. We aimed to investigate surfactant pool as *estimated* by automated postnatal LBC on gastric aspirates without any particular evaluation of surfactant activity or composition. LBC might vary with the relative proportion of efflux of fetal lung fluid mixing with swallowed amniotic fluid and gastric secretions and this could lead to a relative underestimation. Also, it may be difficult to obtain gastric aspirates early from birth for logistic reasons and the postnatal age may affect the reliability of gastric aspirates in representing lung fluid. Therefore, the proportion of fetal lung fluid in gastric aspirates may be influenced by several factors, including clearance/dilution of gastric fluid. Thus, gastric aspirates samples may be biased regarding quantity and quality of surfactant, and these specimens might not equate with the surfactant pool available in the lung. However, we feel that our results are interesting, since they come from a pragmatic study reflecting NICU real life. The pragmatic approach is particularly appropriate for tools or interventions representing refinements of current clinical care [40]. Thus, we used a quick assay to estimate surfactant pool, in a homogeneous population of preterm babies with RDS without any other respiratory disorders and subjected to good perinatal care and a formal, modern, respiratory care protocol. This is representative of the usual neonatal care in developed countries. We cannot evaluate the effect of maternal diabetes or hypothyroidism, as few or no cases presented with these disorders. Nonetheless, we have been able to evaluate all other major clinical factors influencing endogenous surfactant pool and clinical picture of RDS in preterm neonates. It would have been interesting to deeply study the reliability of LBC in different classes of gestational age and, particularly, in extremely preterm neonates who are more likely to need a “guided” surfactant therapy. However, we did not have a large enough population and a preliminary subgroup analysis did not seem to show any difference. This analysis could have been biased and may need to be repeated in larger populations. However, the availability of more accurate tools (such as semiquantitative lung ultrasound and the surfactant adsorption or microbubble tests) reduces the interest in this regard [11, 12, 38, 41]. Finally, ours is a relatively small population but comparable to those of other studies in the field [14].

## Conclusion

Early life LBC on gastric aspirates in CPAP-treated preterm neonates with RDS is significantly influenced by gestational age. LBC is not associated with CPAP failure. Thus, the endogenous surfactant pool available early in life only has a moderate reliability to predict CPAP failure. Further studies are needed to better clarify the biological factors associated to CPAP failure in similar populations.

## Abbreviations

_a_RR: Adjusted relative risks
AUC: Area under the curve
CPAP: Continuous positive airway pressure
FiO_2_: Inspired oxygen fraction
LBC: Lamellar body count
NICU: Neonatal intensive care unit
RDS: Respiratory distress syndrome
ROC: Receiver operating characteristic
SGA: Small for gestational age
